# A Flexible Piezoresistive Sensor Based on ZnO/MWCNTs/PDMS Composite Foam with Overall Performance Trade-Offs

**DOI:** 10.3390/s26051724

**Published:** 2026-03-09

**Authors:** Jun Zheng, Wenting Xu, Wen Ding, Yalong Li, Binyou Xie, Jinhui Xu, Kang Li, Liang Chen, Yan Fan, Songwei Zeng

**Affiliations:** 1 College of Optical, Mechanical and Electrical Engineering, Zhejiang A&F University, Hangzhou 311300, China; zhengjun@zafu.edu.cn (J.Z.); gmalk593968@163.com (W.X.); d2399336513@163.com (W.D.); xie11142000@163.com (B.X.); zafu05110821@163.com (J.X.); likang@zafu.edu.cn (K.L.); 2 School of Physical Science and Technology, Ningbo University, Ningbo 315211, China; liyalong2053@163.com (Y.L.); liang_chen05@126.com (L.C.)

**Keywords:** flexible piezoresistive sensor, wearable strain sensor, ZnO, MWCNTs, human motion monitoring

## Abstract

The flexible foam piezoresistive sensor demonstrates significant potential for wearable strain-sensing applications due to its substantial deformation capacity, excellent flexibility, and cost effectiveness. However, conventional flexible foam piezoresistive sensors often struggle to simultaneously achieve high sensitivity, a wide pressure detection range, fast response and long-term stability. This paper employed a glucose-based sugar-templating method to fabricate a fine-pore (50 μm) foam structure complemented by a dual-filler strategy to enhance overall performance. A robust porous conductive network was constructed by embedding zinc oxide (ZnO) and multi-walled carbon nanotubes (MWCNTs) into a polydimethylsiloxane (PDMS) matrix. The resulting sensor exhibits outstanding piezoresistive properties, featuring a wide linear detection range (0–80% strain) and a high sensitivity of 9.02 kPa^−1^ within the 0–10 kPa pressure range. It demonstrates rapid response/recovery times of 50/70 ms and maintains stable output performance even after 5000 compression cycles at 300 kPa. The sensor also exhibits negligible environmental interference and excellent long-term stability. When attached to finger joints, feet soles, or the throat, the sensor enables functions such as finger bending recognition, race-walking violation discrimination, gait analysis, and vocal fold vibration recognition, thereby demonstrating its considerable potential for application in human–computer interaction and human motion detection.

## 1. Introduction

With excellent flexibility and stability, flexible pressure sensors provide core sensing capabilities for cutting-edge applications such as human–computer interaction, demonstrating its enormous potential in human–computer interaction and human motion detection, and biomedical monitoring [1,2,3,4,5]. Based on their response characteristics, flexible electrical pressure sensors may be categorized as piezoresistive [6], capacitive [7], piezoelectric [8], and triboelectric [9]. Among these categories, piezoresistive pressure sensors have garnered extensive research attention owing to their ability to transduce external mechanical stimuli into quantifiable electrical signals coupled with advantages in fabrication simplicity and cost-effectiveness [10]. In particular, foam-based piezoresistive sensors exhibit prominent advantages, including their light weight, softness, and low-cost nature, coupled with excellent performance in a broad pressure-sensing range and high detection sensitivity [11], rendering them ideal candidates for strain-sensing modules in flexible wearable devices and intelligent robotic systems. Despite these favorable attributes, foam-based piezoresistive sensors still need to satisfy more comprehensive performance requirements in practical applications, including high sensitivity, a wide strain detection range [12], excellent linearity, fast response and recovery dynamics [13,14], and long-term stability. The simultaneous integration of these performance attributes remains a challenge for the current generation of such sensors.

The preparation of conductive fillers, polymer substrates, and structures plays a decisive role in the performance of foam-type piezoresistive sensors. In the field of flexible piezoresistive sensors, the rational design and preparation of conductive fillers, flexible substrates, and device structures are critical to the overall sensing performance [15]. Common conductive fillers include carbon black (CB) [16], graphene oxide (GO) [17], carbon nanotubes (CNTs) [18,19], silver nanowires (AgNWs) [20], multi-walled carbon nanotubes (MWCNTs) [21,22] and polyaniline (PANI) [23,24]. MWCNTs are widely utilized owing to their high electrical conductivity and excellent chemical stability [25]. Common polymer substrates include Ecoflex [26,27], polydimethylsiloxane (PDMS) [28,29], thermoplastic polyurethane (TPU) [30], etc. PDMS has the advantages of adjustable mechanical properties, easy processability and tunability, making it suitable for skin-adherent materials [31,32,33]. Based on the above materials, three mainstream strategies are widely adopted for fabricating flexible conductive foam sensors: the dip-coating method [34], the freeze-drying method [35], and the templating method [36]. For instance, Cai et al. fabricated piezoresistive foam sensors by repeatedly immersing CMF sponges in an AgNWs solution [37]. However, sensors prepared via this approach typically suffer from the risk of conductive filler detachment during cyclic deformation [38,39]. Although Shu et al. improved the interfacial adhesion by modifying the foam surface via dopamine polymerization in solution [40], the thickness and uniformity of the PDA layer are highly sensitive to monomer concentration, reaction temperature, and reaction time, often leading to local over-thickening or incomplete coverage. Freeze-drying is an effective method for preparing highly porous foam sensors. For example, Ye et al. prepared a BC/SA composite aerogel by freeze-drying, and the resulting aerogel exhibited good piezoresistive pressure-sensing performance [41]. Nevertheless, the freeze-drying process is generally complex and time-intensive. In contrast, the template method for preparing porous composite materials features the merits of operational simplicity and safety, in which tunable porosities can be achieved by adjusting the size of solid particles or their mass/volume ratio with polymers matrix [42].

Herein, we report a high-performance sensor fabricated via a glucose-templating technique to create a microporous (50 μm) foam integrated with a synergistic dual-filler system of ZnO and MWCNTs within a polydimethylsiloxane (PDMS) matrix. This structure enables the MWCNTs and ZnO on the foam wall to be conveniently interconnected, resulting in a broad linear range (0–80%), high sensitivity (9.02 kPa^−1^ at 0–10 kPa), and rapid response/recovery times (50/70 ms). Notably, the device retains stable performance after 5000 cycles under 300 kPa loading and exhibits robustness in the face of environmental fluctuations in temperature and humidity. These advantages, combined with a simple fabrication process and favorable softness, indicate the sensor’s great potential in gait detection and promising application prospects in related fields such as vocal cord vibration recognition and human motion monitoring.

## 2. Experimental Section

### 2.1. Materials

The PDMS base and curing agent were purchased from Dow Corning (Sylgard 184, Midland, MI, USA). MWCNTs (diameter = 3–15 nm, length = 15–30 μm) were purchased from Suiheng Graphene Technology (Shenzhen, China). Zinc oxide (ZnO) nanoparticles were supplied by Shanghai Aladdin Biochemical Technology Co., Ltd. (6th Floor, Sanda Building, 1 Xinjinqiao Road, Pudong New Area, Shanghai, China). Glucose particles were acquired from Macklin Reagent (Shanghai, China).

### 2.2. ZnO/MWCNTs/PDMS Fabrication of the Sensor

**Figure 1:**
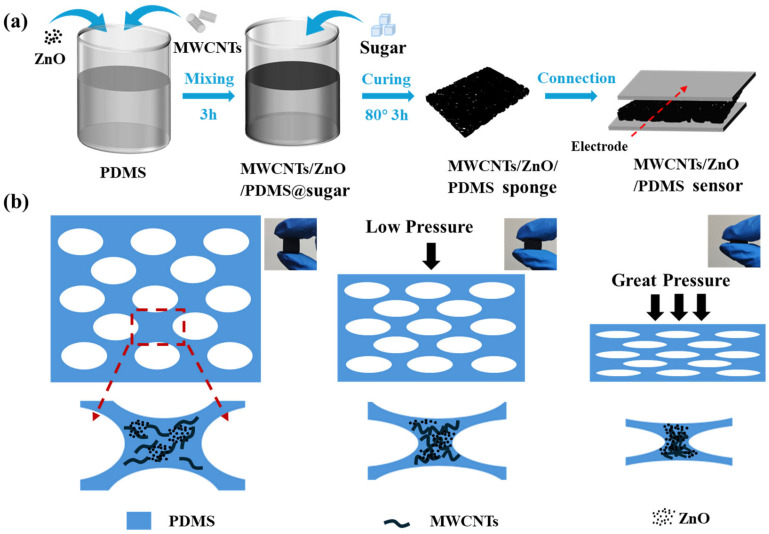
Schematics of (**a**) the fabrication process and (**b**) the sensing mechanism for the ZMP flexible piezoresistive sensor. The content between the red arrows in (**b**) represents the enlarged portion within the red box.

The sensor was fabricated using the sugar template method, as illustrated in Figure 1a. The detailed procedure is described as follows: First, the PDMS base and curing agent were weighed at a 10:1 mass ratio in a clean beaker and gently stirred for 2 min to facilitate the removal of entrapped air bubbles. Subsequently, MWCNTs (4 wt%) and ZnO nanoparticles (0.6 wt%) were sequentially added to the matrix. The mixture was subjected to magnetic stirring at 600 rpm for 3 h to achieve homogeneous dispersion, resulting in a gray–black conductive prepolymer. Next, glucose particles with different diameters (50 µm, 200 µm, 400 µm) were added into the beaker at a PDMS-to-glucose mass ratio of 4:1. Stirring was continued for 5 min to ensure complete coating of the sugar particles with the prepolymer. The mixture was then poured into a custom-made polytetrafluoroethylene (PTFE) mold (cavity dimensions: 1.5 cm × 1.5 cm × 1.5 cm), gently vibrated for 30 s to eliminate residual bubbles, and transferred to an oven at 80 °C for 3 h for curing. After cooling to room temperature, the cured elastomer was demolded and cut into small pieces (15 mm × 10 mm × 3 mm) using a knife. The samples were immersed in deionized water at 60 °C for 12 h, which was followed by drying in a 60 °C oven; this process was repeated twice to completely dissolve the sugar template. The resulting ZnO/MWCNTs/PDMS composite foams were labeled as ZMP, ZMP 1, and ZMP 2, respectively, according to the glucose particle size used. Finally, conductive silver fabric electrodes were attached to both ends of the foam with conductive silver paste, thereby fabricating the ZMP flexible piezoresistive sensor. The sensors used in the subsequent measurements were 10 mm × 10 mm × 10 mm cubes fabricated with the optimal composition (4 wt% MWCNTs and 0.6 wt% ZnO), possessing a bulk resistance of 3200 Ω (Supplementary Figure S1 and Table S1).

As demonstrated in Figure 1b, within the three-dimensional ZMP foam, MWCNTs are uniformly embedded in the elastic PDMS matrix in a network form, constructing a conductive skeleton. ZnO nanoparticles are incorporated to enhance the mechanical strength of the foam [41]. When an external mechanical load is applied, the PDMS skeleton first undergoes reversible compression, leading to a rapid reduction in the spacing between pore walls. Consequently, the MWCNTs, which were initially separated by an insulating elastic regions, are brought into closer proximity due to the deformation, establishing new conductive pathways. This inter-contact among MWCNTs inside the PDMS matrix increases the number of effective conductive channels, thereby reducing the overall electrical resistance.

### 2.3. Measurement Instruments

SEM (ZEISS Sigma 300, Carl Zeiss AG, Oberkochen, Germany) was used to characterize the cross-sectional morphology of the sensor. An electrochemical workstation (CHI760e, Shanghai Chenhua Instrument Co., Ltd., Hong Kong, China) was used to test the sensor’s response and recovery times. A piezometer 6374 (KEMBLEY, Cleveland, OH, USA) and a testing machine (Mark 10) were used to test the stress–strain curves of the foam as well as the resistance changes and stability of the sensor under different pressures. An oscilloscope DS4034 (Jetyoo Industrial, Shanghai, China) and a voltmeter MPS-3303 (Shenzhen Maiwei Instrument Co., Ltd., Shenzhen, China) were used to test the time differences between walking and running.

## 3. Results

### 3.1. Characterization

**Figure 2:**
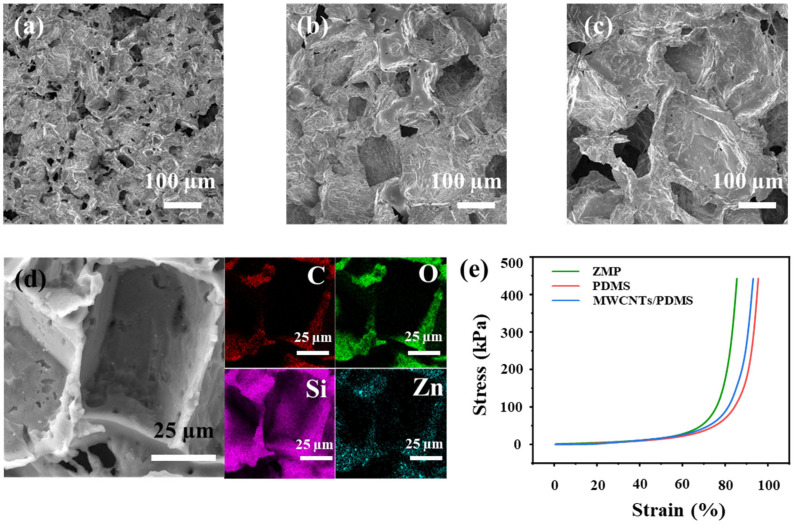
(**a**) SEM image of ZMP, (**b**) ZMP 1, and (**c**) ZMP 2 at a magnification of 100×. (**d**) EDS image of the ZMP material distribution. (**e**) Stress–strain curves of PDMS foam, MWCNTs/PDMS foam, and ZMP foam.

As illustrated in Figure 2, Field Emission Scanning Electron Microscope (FE-SEM) images confirm the porous structure of the ZMP composite foams. The pore size can be effectively tuned by the choice of the sugar template. The ZMP, ZMP 1, and ZMP 2 foams (Figure 2a–c) exhibit average pore sizes of approximately 50 µm, 200 µm, and 400 µm, respectively. Elemental distribution was analyzed by energy-dispersive X-ray spectroscopy (EDS). The surface mapping in Figure 2d reveals a homogeneous dispersion of the constituent elements (C, O, Si, and Zn) throughout the foam matrix. Carbon originates primarily from the MWCNTs and the PDMS backbone; oxygen and silicon correspond to the Si-O-Si chains of PDMS; and zinc is distributed at the nanoscale, which is consistent with the incorporated ZnO nanoparticles. The high uniformity of element distribution ensures the consistency of the conductive network throughout the three-dimensional space, providing the microstructural foundation for the device’s wide sensing range, high sensitivity, and long-term stability. As demonstrated in Figure 2e, the ZMP sensor exhibits minimal deformation under identical pressure, which is a result of the rigidity enhanced by the incorporated MWCNTs and ZnO. The incorporation of MWCNTs imparts excellent electrical conductivity to the composite (Supplementary Figure S4), while MWCNTs and ZnO enhance the mechanical strength of the foam, thereby mitigating fragmentation and structural damage. The stress–strain relationship and the relative resistance change rate of the ZMP sensor are related to the foam pore size [43,44] (Supplementary Figures S2, S3 and S5).

### 3.2. Performance Analysis

**Figure 3:**
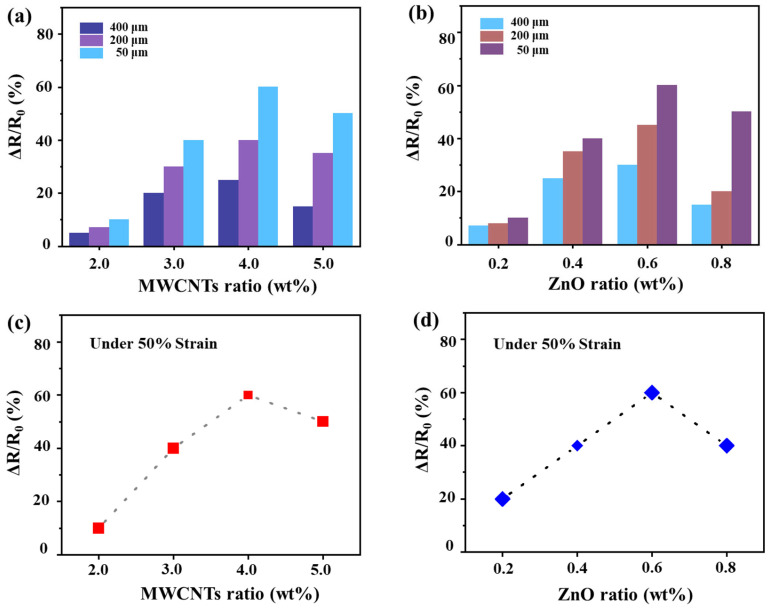
(**a**) Resistance change rate at 50% strain with ZnO weight ratio fixed at 0.6 wt% and different MWCNTs contents under different pore sizes (50 μm, 200 μm, 400 μm). (**b**) Resistance change rate at 50% strain with MWCNTs weight ratio fixed at 4 wt% and different ZnO contents under different pore sizes (50 μm, 200 μm, 400 μm). (**c**) At 50% strain, the resistance change rate of sensors with a fixed ZnO mass fraction (0.6 wt%) and varying MWCNTs mass fractions. (**d**) At 50% strain, the resistance change rate of sensors with a fixed MWCNTs mass fraction (4 wt%) and varying ZnO mass fractions.

For ZMP sensors, their performance depends on multiple factors, such as the ratio of MWCNTs to ZnO and the pore size of the foam. Research is required to determine the optimal parameters for achieving superior sensor performance. As illustrated in Figure 3a, with the ZnO ratio fixed at 0.6 wt%, samples with varying MWCNTs mass fractions (2 wt%, 3 wt%, 4 wt%, and 5 wt%) were fabricated. For each MWCNTs concentration, different pore sizes (50 μm, 200 μm, 400 μm) were adopted, and the resistance change rate of each sample group was measured under 50% strain. It is observed that the sensor with a pore size of 50 μm exhibits a significantly higher resistance change rate compared to those with 200 μm and 400 μm pores. With the ZnO concentration fixed at 0.6 wt%, the relationship between the resistance change rate of sensors with different foam pore diameters and the variation in MWCNTs content exhibits a similar trend under 50% deformation with the maximum resistance change rate attained when the MWCNTs content is 4 wt%. Specifically, the relationship between the resistance change rate of the sensor with a 50 μm foam pore diameter and the MWCNTs content is illustrated in (Figure 3c). Similarly, as demonstrated in Figure 3b, with the MWCNTs ratio fixed at 4 wt%, samples were prepared with different ZnO concentrations (0.2 wt%, 0.4 wt%, 0.6 wt%, and 0.8 wt%). For each concentration, samples with different pore sizes (50 μm, 200 μm, 400 μm) were selected to measure the resistance change rate of each group of samples under 50% strain. The results indicate that sensors with a pore size of 50 μm exhibit significantly higher resistance change rates compared to those with pore sizes of 200 μm and 400 μm. Under 50% strain and with the MWCNTs content fixed at 4 wt%, the resistance change rate across different foam pore sizes follows a similar trend with varying ZnO content, peaking at a ZnO concentration of 0.6 wt%. The situation of the sensor whose pore size is 50 μm is demonstrated in Figure 3d.

In summary, sensors fabricated with 4 wt% MWCNTs, 0.6 wt% ZnO, and a pore size of approximately 50 μm exhibit optimal piezoresistive performance. This composition establishes an ideal balance between conductive network connectivity and the number of tunneling junctions. Any further increase in filler content induces particle aggregation, which raises local stiffness, impedes stress transfer, and ultimately reduces sensitivity. Therefore, all subsequent tests were based on this optimized sensor (4 wt% MWCNTs, 0.6 wt% ZnO, 50 μm pore size).

### 3.3. Piezoresistive Performance

**Figure 4:**
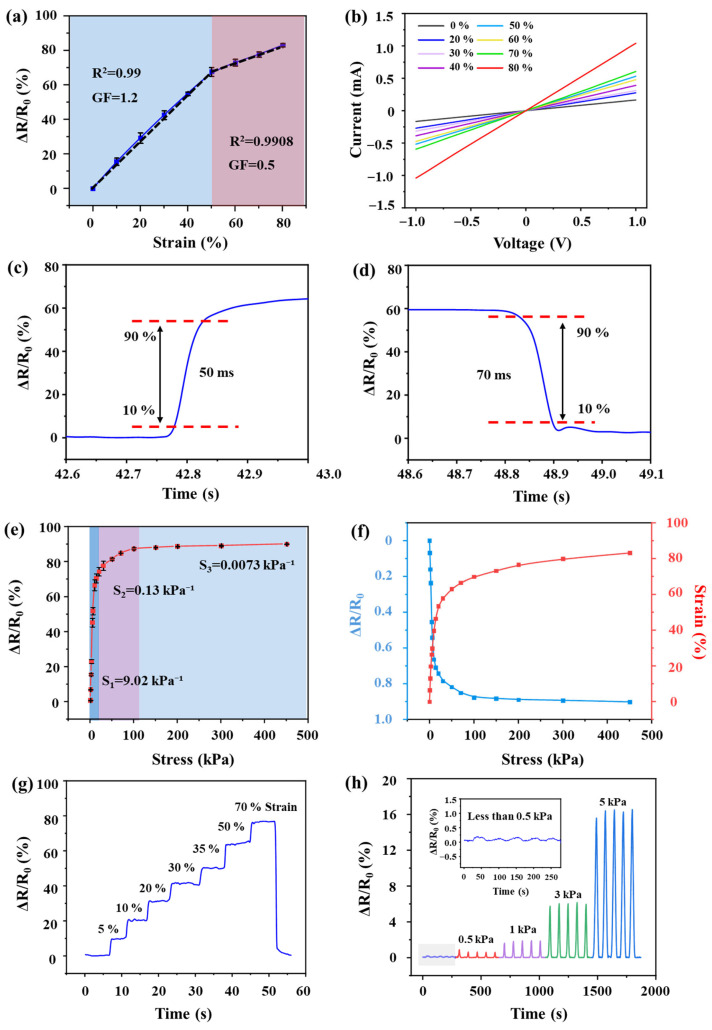
Strain and relative resistance change curves of ZMP (**a**), The dashed line in the figure represents the fitted curve, while the solid line depicts the strain versus relative resistance variation curve of the sensor. IV curves and sensitivity curves (**b**). Response of ZMP under 50% strain (**c**) and recovery time (**d**). (**e**) Sensitivity curve of ZMP in the range of 0–450 kPa. (**f**) The relationship between strain (ε) and sensitivity (S). (**g**) Resistance change rate of ZMP under continuous compressive strain in the range of 0–70%. (**h**) Minimum detection limit of ZMP sensors. The grey-shaded area represents pressure responses below 0.5 kPa, with this section enlarged in the diagram.

As shown in Figure 4a, the linear sensing range of the ZMP sensor is divided into two distinct regions with gauge factor (GF) values of 1.2 (strain ε = 0–50%) and 0.5 (ε = 50–80%), respectively. The GF values, calculated using the formula $GF=\left(∆R/R_{0}\right)/\varepsilon$, are distributed closely around the fitted values with minor fluctuations, indicating good linear sensing behavior. Figure 4b presents the I–V curves of the device under different static pressures. All curves exhibit good linear ohmic characteristics, with the slope increasing monotonically as pressure increases, indicating the formation of a stable three-dimensional conductive network among MWCNTs, PDMS, and ZnO. This provides a reliable foundation for subsequent high-precision signal decoding. The dynamic response performance of the sensor is further illustrated in Figure 4c,d. Under 50% strain, the device has a fast response time of 50 ms and a recovery time of 70 ms, surpassing most of the reported pure PDMS-based piezoresistive sensors. Figure 4e systematically presents the sensitivity curve of the ZMP foam sensor across a wide pressure range. Sensitivity (S) is calculated as $S=\left(∆R/R_{0}\right)/∆P$, where ΔR/R_0_ is the relative resistance change and ΔP is the applied pressure change. The curve exhibits three distinct linear regions: a high sensitivity of 9.02 kPa^−1^ in the low-pressure range (0–10 kPa), a moderate sensitivity of approximately 0.13 kPa^−1^ in the medium-pressure range (10–100 kPa), and a low sensitivity of 0.0073 kPa^−1^ at high pressures (100–450 kPa). This trend can be attributed to the rapid contact establishment between conductive pathways in the foam’s cell walls during initial compression, leading to a sharp resistance drop. At high pressures, further pore compaction causes the number of conductive paths to approach saturation, stabilizing the resistance change and thus ensuring detectable signal resolution over a wide range. The relationship between strain (ε) and sensitivity (S) is shown in Figure 4f. It is demonstrated that the foam shows linear strain within 50 kPa; at this time, the resistance variation rate rises sharply. Sensitivity $S=\left(∆R/R_{0}\right)/∆P$; therefore, the sensitivity is extremely high in this pressure range. With the increase in pressure, the foam is gradually compressed and compacted. At this time, the deformation decreases, the resistance change rate decreases, and the S value also decreases. Figure 4g demonstrates the relative resistance change in the sensor under continuously increasing strain from 0 to 70%. The relative resistance change rises monotonically from 10% to 78% as strain increases from 5% to 70%, indicating the sensor’s suitability for a wide spectrum of physiological and motion monitoring scenarios. The detection limit of the sensor is 0.5 kPa, as displayed in Figure 4h. This low detection threshold is attributed the use of fine-grained glucose instead of traditional sugar as the sugar template in this study. This choice resulted in a foam with a finer pore structure, thereby enabling MWCNTs and ZnO on the pore walls to interconnect even under low pressure. This interconnected network significantly enhances the sensor’s sensitivity.

**Figure 5:**
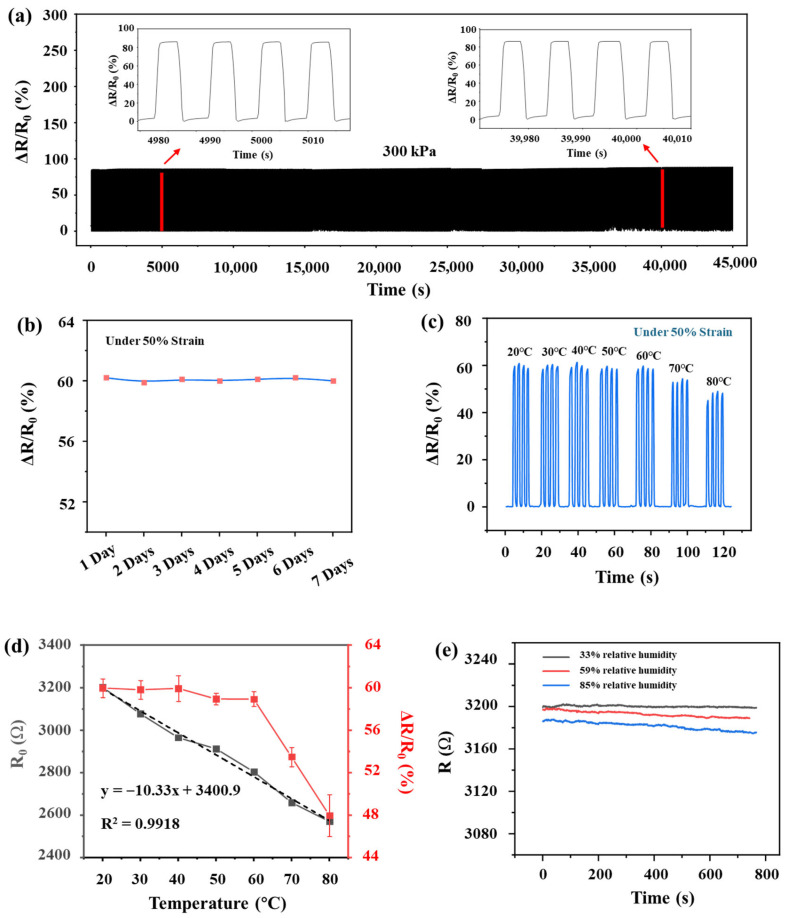
(**a**) Stability test, 5000 compression cycles under 300 kPa high pressure. The magnified images shown in the figure indicate the sensor’s test cycle stability at 5000 s and 40,000 s, respectively. (**b**) Stability of the sensor over a week. (**c**) Rate of resistance change at 50% strain for ZMP sensors at different temperatures. (**d**) Initial resistance of ZMP sensors at different temperatures and rate of resistance change under 50% strain, The dashed line in the figure represents the fitted curve. (**e**) Initial resistance variation in ZMP sensors under continuous exposure to 33%, 59%, and 85% relative humidity at 25 °C.

In practical applications, long-term stability emerges as a critical determinant of sensor efficacy and durability. Durability tests of this sensor are illustrated in Figure 5a. After 5000 consecutive compression cycles at 300 kPa, the sensor retains nearly identical sensitivity, baseline resistance, and hysteresis compared to its initial state. To evaluate the sensor’s long-term stability, its performance was assessed over a seven-day period by applying a consistent mechanical load daily and measuring the corresponding change in sensor resistance. As illustrated in Figure 5b, the resistance change rate remained stable with minimal fluctuation throughout the testing period. The resistance change rate (ΔR/R0) of the sensor was evaluated under a fixed strain of 50% across a temperature range of 20–80 °C. The results in Figure 5c indicate that the sensor’s piezoresistive response remains remarkably stable within the 20–60 °C regime. However, an attenuation in sensitivity is observed when the temperature exceeds 60 °C. Despite this high-temperature degradation, the sensor retains full operational functionality for its intended application in human motion detection, as ambient and skin temperatures in such scenarios typically remain well below the 60 °C threshold. As illustrated in Figure 5d, the foam sensor exhibits a distinct Negative Temperature Coefficient (NTC) behavior. The initial resistance (R0) demonstrates a highly linear response to temperature variations with good linear fitting (correlation coefficient R^2^ = 0.9918). Within the range of 20–80 °C, the fractional change in resistance per degree, denoted as α ($\alpha =\frac{1}{R}\frac{dR}{dT}$), is calculated to be approximately −0.32%·°C^−1^, indicating that the sensor possesses low temperature sensitivity. At 50% strain, the sensor exhibits stable piezoresistive responses (ΔR/R0) from 20 to 60 °C, owing to the resistance reduction induced by the mechanical compression of the foam substantially outweighing the thermal effects. Above 60 °C, ΔR/R0 decreases due to thermally activated carrier generation, which lowers the contact barrier sensitivity and weakens strain-induced resistance change. By utilizing fine-grained glucose templating to create a microporous structure, we established a highly interconnected MWCNT/ZnO network. This design ensures that mechanically induced resistance changes significantly surpass thermal fluctuations, thereby improving the sensor’s insensitivity to temperature variations and optimizing its piezoresistive characteristics. The influence of humidity on sensor performance is presented in Figure 5e. Controlled humidity environments were established using saturated salt solutions of magnesium chloride (MgCl_2_), sodium bromide (NaBr), and potassium chloride (KCl), which maintain relative humidities (RH) of 33%, 59%, and 85%, respectively, at room temperature (25 °C). It is shown that the sensor’s resistance remains virtually invariant at 33% RH. The total resistance drift over an 800 s interval is only 0.29% and 0.43% respectively, at 59% and 85% RH. It demonstrates that the sensor exhibits exceptional stability against humidity interference. This robustness is attributed to the use of the glucose template method, where the high solubility of glucose ensures its thorough elimination, yielding a pristine and uniform porous network within the PDMS matrix. This optimized microstructure improves structural uniformity, resulting in better overall performance. For a detailed comparison with several recently reported advanced foam-based sensors, including the material type, GF, response/recovery time, strain range, and stability, refer to Table 1.

### 3.4. Application

**Figure 6:**
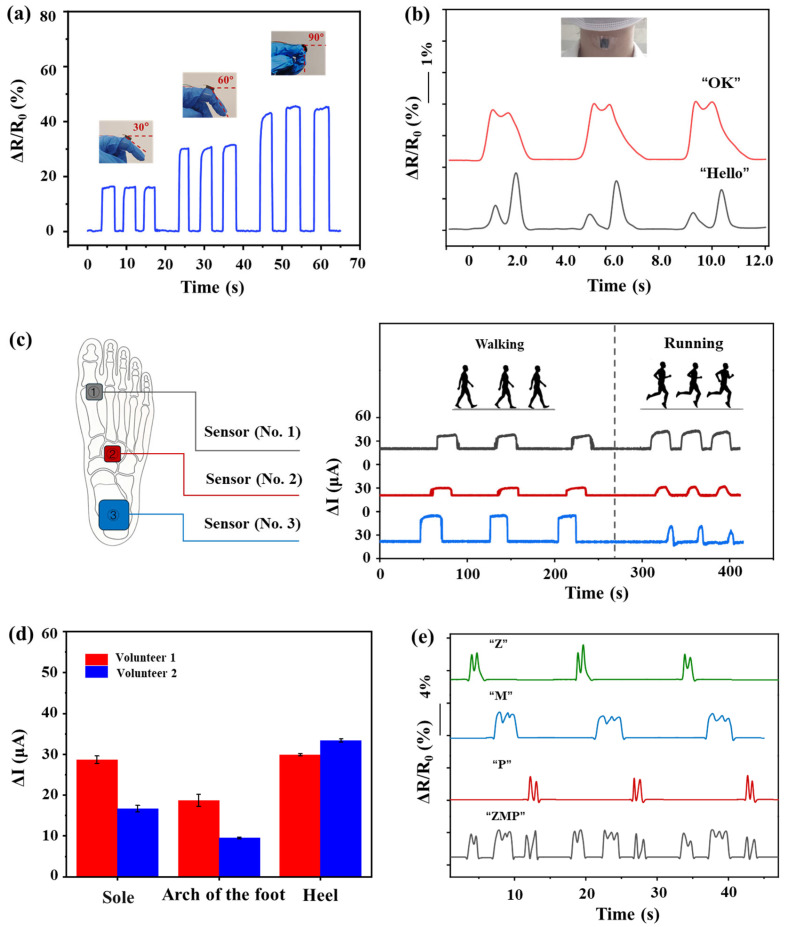
(**a**) Response signals of the sensor under varying finger-bending angles. (**b**) Vocal cord vibration monitoring via a throat = attached sensor during the utterance of ‘OK’ and ‘Hello’. (**c**) A sensor attached to the forefoot, midfoot, and heel areas of a sports shoe to monitor movements at each location and time differences. (**d**) Monitoring the plantar force exerted by two different volunteers. (**e**) Placing the sensor on a desk and writing the letters ‘Z’, ‘M’, ‘P’, and then the continuous letters ‘ZMP’ on the sensor surface with a pen.

Due to its favorable response across a broad pressure range, the sensor exhibits extensive applicability in practical scenarios. As shown in Figure 6a, a foam piece (15 mm × 10 mm × 3 mm) was attached to the dorsal side of the proximal phalanx of the right index finger using double-sided tape. When the finger was bent to 30°, 60°, and 90°, the sensor’s relative resistance increased in clear, stepwise increments, demonstrating its ability to distinguish joint angles in real time, which is promising for gesture recognition and rehabilitation training.

The sensor is also capable of detecting subtle strains. As illustrated in Figure 6b, the sensor was mounted on a volunteer’s throat to monitor vocal cord vibrations in real time. When the volunteer spoke the words ‘Hello’ and ‘OK’ at normal volume, the resulting vocal vibrations induced minute deformations in the foam. These deformations were converted into electrical signals by the sensor and subsequently recorded using a picoammeter (KEITHLEY, Model 6374, Cleveland, OH, USA).

The sensor’s high sensitivity to subtle pressure variations enables effective motion detection. As the soles of the feet are among the most dynamically loaded regions of the body, ZMP sensors were placed at distinct plantar locations (Figure 6c) to capture different motion states, such as walking and running. Three sensors of the same specifications (labeled as No. 1 to No. 3) were connected in a parallel circuit powered by a 2 V DC source, each in series with a 51 kΩ current limiting resistor. Voltage changes across the resistors were recorded with an oscilloscope, and the corresponding current variations were calculated using $△I=\left(U-U_{o}\right)/R_{r}$, where U is the measured voltage, U_0_ is the initial voltage, and the R_r_ is 51 kΩ. The sensors were fixed at the forefoot (No. 1), the midfoot (No. 2), and the heel (No .3). Measurements were taken during walking and running. During walking, the heel typically contacts the ground first, which is followed by a rolling motion through the midfoot, and finally a push off from the forefoot. Accordingly, in the sensor array, the heel-mounted sensor (No. 3) responds first, which is followed sequentially by the midfoot sensor (No. 2) and the forefoot sensor (No. 1). The rate of resistance change is highest at the heel (sensor 3), which is followed by the forefoot (sensor 1). The midfoot sensor (No. 2) exhibits the weakest signal as a result of the limited ground contact occurring in the arch region during the walking cycle. In running, particularly under professional training conditions, a forefoot-first ground-contact pattern is typically observed. The forefoot strikes the ground with greater force, causing the corresponding sensor (No. 1) to respond first and exhibit both the highest rate of resistance change and the longest signal duration. The contact then progresses sequentially through the midfoot (sensor No. 2) and finally to the heel (sensor No. 3), with each sensor generating a corresponding signal in turn. This pronounced signal differentiation can be leveraged for the real-time, accurate detection of posture-related fouls in race-walking competitions, offering a viable alternative to conventional approaches such as manual adjudication or video-assisted review. Furthermore, the combined signal output from this three-sensor array can also be utilized to monitor individual gait patterns, as shown in Figure 6d.

The ZMP sensor further demonstrates handwriting recognition capability. As displayed in Figure 6e, when the sensor is placed on a desktop and the letters ‘Z’, ‘M’, and ‘P’ are written sequentially on its surface with a pen, distinct response profiles are generated. These signals exhibit strong consistency across three consecutive writing cycles. Moreover, continuous writing of the sequence ‘ZMP’ over three repeated cycles confirms the sensor’s reliable and stable performance under periodic mechanical input.

## 4. Conclusions

This paper proposes a simple, low-cost sugar template method to construct a ZMP composite foam piezoresistive sensor featuring a three-dimensional porous structure. Sparsely distributed ZnO particles enhance the foam’s overall mechanical properties, while MWCNTs form three-dimensional conductive pathways enabling rapid charge migration. For preparation, finely particulate glucose was employed as the sugar template instead of conventional granular sugar. Its incorporation into the PDMS composite promoted the formation of pores with a more uniform distribution and finer size. This structural refinement enables the MWCNTs on the foam walls to interconnect even under low applied pressure, thereby significantly enhancing the sensor’s sensitivity. These optimizations in the foam architecture consequently improve the overall performance of the sensor. In terms of performance, the sensor exhibits a sensitivity of 9.02 kPa^−1^ at low pressure (0–10 kPa), 0.13 kPa^−1^ at medium-to-high pressures (10–100 kPa), and remains stable at 0.0073 kPa^−1^ across the high-pressure range (100–450 kPa). Response/recovery time is 50/70 milliseconds with performance remaining stable after 5000 compression cycles at 300 kPa. Furthermore, by adjusting aperture and filling ratio, the sensor can detect throat vibrations and heavy-load gait patterns, distinguish finger bending angles, and recognize distinct stress waveforms during writing. It demonstrates significant potential in applications such as flexible wearables, intelligent robotics, and assistive communication systems.

## Figures and Tables

**Table 1:** A comparison of the reported foam-based piezoresistive sensors with the present work.

Material	GF	Response/Recovery (ms)	Strain Range (%)	Cycles	Ref.
NR/PEDOT: PSS	0.983	140/120	70	1000	[45]
NOC PPP	6.6619	120/100	70	1000	[46]
Vc-TPU/CNT@AgNPs	1.4	20/25	90	500	[47]
POE/CNS	1.03	168	90	20,000	[48]
CB/PDMS	1.12	45	91	15,000	[49]
PEBA/CNS	1.24		70	1000	[50]
CNT/SRFs	0.83	150/150	80	39,000	[23]
AgNTs@GrF	1.12	126/5000	90	3000	[51]
RGO-CNT/Melamine	1.6	19.3	80	11,000	[52]
CNT/TPU	1.22		70	2000	[53]
PDMS-cPFF	−20.5		40	1000	[54]
MWCNTs/PU	1.68	600/500	80	2000	[55]
rGO-CNT/PDA@PDMS	2.13	260/640	60	500	[56]
EVA/LDPE/MWCNTs	3.9	170/190	60	1000	[57]
BC/SA	14.27	112/154	70	1000	[41]
ZnO/MWCNTs/PDMS	1.2	50/70	80	5000	This work

## Data Availability

Data will be made available on request.

## Supplementary Materials

The following supporting information can be downloaded at https://www.mdpi.com/article/10.3390/s26051724/s1, Figure S1: The same batch of sensors was cut into nine pieces. Table S1: The resistance of each resistor measured with a multimeter is approximately as shown in the table below. Figure S2: Foam structures formed by sugar moulds of varying dimensions. Figure S3: Stress-strain curves of ZMP, ZMP 1, and ZMP 2. Figure S4: Strain and relative resistance change curves of ZMP, MWCNTs/PDMS, and PDMS. Figure S5: Strain and relative resistance change curves of ZMP, ZMP 1, and ZMP 2.
