# Supplementary material for: A Flexible Piezoresistive Sensor Based on ZnO/MWCNTs/PDMS Composite Foam with Overall Performance Trade-Offs
Source: Sensors (Basel). 2026 Mar 9;26(5):1724. doi: 10.3390/s26051724 (PMC12986935; doi:10.3390/s26051724)
Supplement: Supplementary file 1 [file sensors-26-01724-s001.zip › sensors-4114306-supplementary.pdf]

Supplementary Information

A Flexible Piezoresistive Sensor Based on ZnO/MWCNTs/PDMS Composite Foam with Overall Performance Trade-offs

Jun Zheng[a,1], Wenting Xu[a,1], Wen Ding[a], Yalong Li[b], Binyou Xie[a], Jin hui Xu[a],Kang Li[a] Liang Chen[b], Yan Fan[a] and Songwei Zeng[a*]*

[a]*College of Optical, Mechanical and Electrical Engineering, Zhejiang A&F University, Hangzhou 311300, China*

[b]*School of Physical Science and Technology, Ningbo University, Ningbo 315211, China*

[1] *Jun Zheng and Wenting Xu contributed equally to this work.*

** Correspondence authors:*

E-*mail addresses: fanyan503@zafu.edu.cn* (Yan Fan); *zsw@zafu.edu.cn* (Songwei Zeng).

Contents

Figure S1. The same batch of sensors was cut into nine pieces. S3

Table S1. The resistance of each resistor measured with a multimeter is approximately as shown in the table below. S3

Figure S2. Foam structures formed by sugar moulds of varying dimensions S3

Figure S3. Stress-strain curves of ZMP, ZMP 1, and ZMP 2 .... S4

Figure S4. Strain and relative resistance change curves of ZMP, MWCNTs/PDMS, and PDMS. S5

Figure S5. Strain and relative resistance change curves of ZMP, ZMP 1, and ZMP 2. .... S5

References. S6

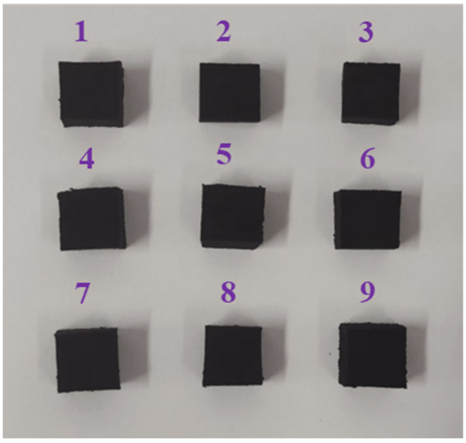

Figure S1. The same batch of sensors was cut into nine pieces.

We prepared a batch of samples according to the optimal ratio (4 wt% MWCNTs and 0.6 wt% ZnO) and cut them into 9 cubes measuring 1 cm × 1 cm × 1 cm. Subsequently, the bulk resistance of each cube was measured, and the results are shown in **Table S1**. The mean bulk resistance of this batch of samples was determined to be 4046 Ω. After adding conductive silver paste as the electrode, the average initial resistance of the foam sensor is 3200 Ω.

Table S1. The bulk resistance of foam cubes

**Number**	**1**	**2**	**3**	**4**	**5**	**6**	**7**	**8**	**9**
**Resistance(Ω)**	4482	6405	2893	5093	3992	5292	2229	3245	2787

The reason why the foam resistance is higher than the sensor resistance is that conductive silver paste was used when connecting the electrodes. After connecting the electrodes, the sensor resistance stabilizes around 3200 Ω.

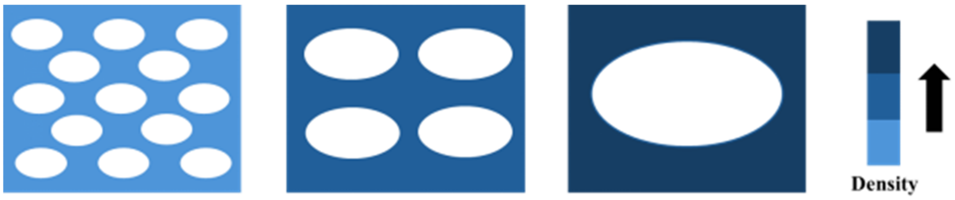

Figure S2. Foam structures formed by sugar moulds of varying dimensions

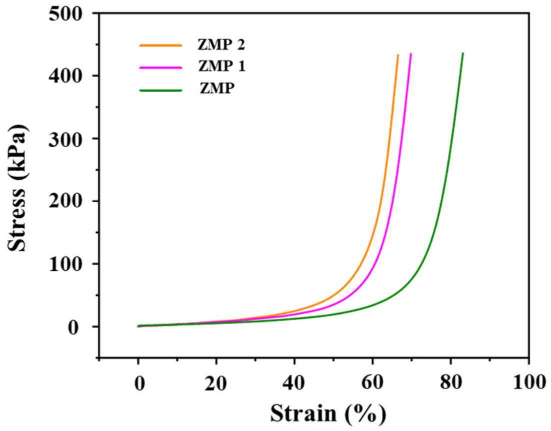

Figure S3. Stress-strain curves of ZMP, ZMP 1, and ZMP 2

We tested the stress-strain curves of ZMP1 and ZMP2 using a press. From the results, it can be seen that compared to ZMP, ZMP1 and ZMP2 have smaller deformation under the same pressure. We agree that the larger the pore diameter of the foam sensor prepared by the sugar template method, the greater the deformation of the sensor under the same conditions [43]. The abnormal conclusion of the sensor in our experiment is mainly based on the following considerations: the deformation of the foam sensor depends on the combined effect of the foam pore size, porosity, pore dispersion uniformity. When other conditions are the same, the larger the pore diameter or porosity, the greater the deformation of the foam sensor. However, we fixed the mass of PDMS, MWCNT, ZnO and sugar when preparing foam with different pore sizes. All samples were prepared in a mold with a size of $3 \times 3 \times 3$ cm^3. Therefore, foam with smaller pore size (ZMP, 50μm pore size) has more uniform pore distribution, more pores and higher porosity, which enables it to deform under lower pressure and show higher resistance change rate in the low pressure range. For foam sensors with larger aperture (such as ZMP2, 400 μm pore size), the number of holes is less, and it is more difficult to achieve uniform distribution; Meanwhile, the density of PDMS, MWCNT, and ZnO mixture is relatively high, and the increase in density makes it difficult to undergo deformation, as shown in the **Figure S2.** Therefore, as shown in the **Figure S3**, when the same pressure is applied, the deformation of ZMP2 is the smallest, while the deformation of ZMP is the largest.

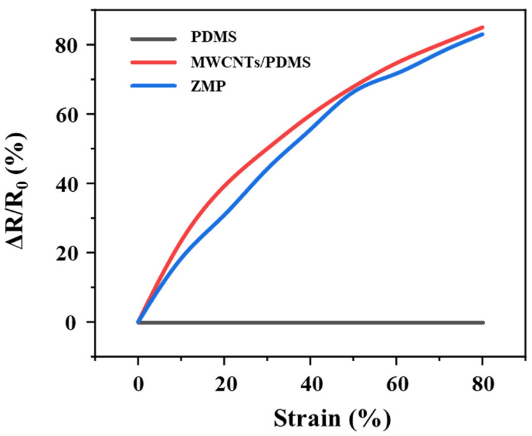

Figure S4. Strain and relative resistance change curves of ZMP, MWCNTs/PDMS, and PDMS.

The incorporation of ZnO and MWCNT increases the stiffness compared to the original elastomer. Although the resistance change rate of WMCNTs/PDMS is higher than that of ZMP, in comparison, ZMP has better linearity after adding ZnO, the rigidity of the foam increases, reducing the resistance change rate under the same strain. As seen in **Figure S3**, compared with WMCNTs/PDMS, ZMP is more linear.

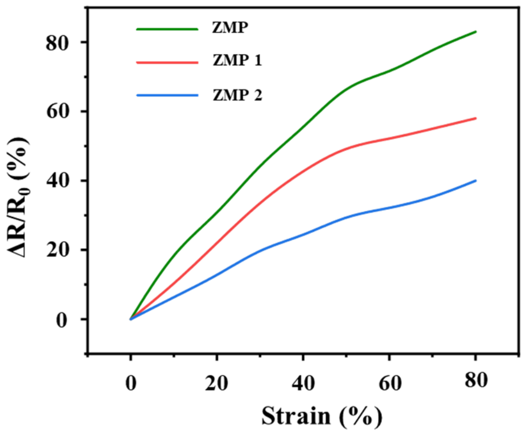

Figure S5. Strain and relative resistance change curves of ZMP, ZMP 1, and ZMP 2.

It is recognized that the influence of porosity on the performance of foam sensor is that under other conditions, the higher the porosity, the greater the deformation of the sensor, but the higher the porosity will lead to non-linear resistance change and poor stability [44]. In our sugar-template method (as mentioned in the answer to the previous question), porosity is not the only parameter that determines the deformation or piezoresistive performance of the sensor. The distribution of large-sized sugar particles

is generally poor, and there is randomness in SEM estimation of porosity, so the influence of porosity on deformation or resistance change rate is not strongly correlated. The relationship between the resistance change rate and deformation of our sensor is shown in **Figure S5**. Under the same deformation, the ZMP resistance change rate of small aperture foam sensor is the highest.

References

43. Elizabeth, I.; Athira, C.; Barshilia, H.C. Influence of Pore Size on the Piezoresistive Behavior of CNT/PDMS Sponge Sensors. *Discov. Sens.* **2025**, *1*, 13, doi:10.1007/s44397-025-00013-1.
44. Herren, B.; Webster, V.; Davidson, E.; Saha, M.C.; Altan, M.C.; Liu, Y. PDMS Sponges with Embedded Carbon Nanotubes as Piezoresistive Sensors for Human Motion Detection. *Nanomaterials* **2021**, *11*, 1740, doi:10.3390/nano11071740.
